# Combine effects of robotic assisted gait training with transcranial magnetic stimulation on gait metrics and balance in stroke patients: a pilot randomized control

**DOI:** 10.3389/fneur.2025.1655409

**Published:** 2025-11-20

**Authors:** Abdullah Jehangir, Irfan Ahmad, Yali Feng, Wang Wei Juan, Ting Chen, Ying Yin

**Affiliations:** 1 Department of Rehabilitation Medicine, Second Affiliated Hospital of Chongqing Medical University, Chongqing, China; 2 Department of Radiology, Second Affiliated Hospital of Chongqing Medical University, Chongqing, China

**Keywords:** robotic assisted gait training (RAGT), trans-cranial magnetic stimulation (TMS), stroke rehabilitation, gait recovery, postural balance, hemiparesis, motor cortex excitability, interhemispheric inhibition

## Abstract

**Background and objective:**

Stroke often causes gait and balance impairments due to disrupted neural control. While robotic-assisted gait training (RAGT) improves motor function, combining it with low-frequency transcranial magnetic stimulation (LF-rTMS) may enhance neuroplasticity and recovery. This pilot RCT investigates the feasibility and synergistic effects of RAGT + LF-rTMS on gait and balance in stroke patients.

**Materials and methods:**

This pilot RCT included 21 stroke patients randomized into three groups: RAGT + active LF-rTMS, RAGT + sham rTMS, and control (standard physiotherapy). RAGT used an exoskeleton with adjustable speed (0.8–1.8 km/h) and body-weight support (40–60%). LF-rTMS (1 Hz, 80% RMT) targeted the unaffected hemisphere’s M1. Outcomes included 3D gait analysis (spatiotemporal metrics), dynamic balance (COP sway), and clinical scales (FMA-LE, BBS, MMT).

**Results:**

The RAGT+TMS group demonstrated more improvements in balance (BBS: Δ22.58 vs. Δ15.40 in RAGT+sham TMS; *p* = 0.05) and motor function (FMA: Δ5.86 vs. Δ1.61; *p* = 0.04) compared to other groups. Gait analysis revealed significant left step length increases in RAGT+TMS (*Δ*6.86 cm, *p* = 0.04), while balance metrics showed reduced postural sway (oscillation length: Δ − 25.01 cm, *p* = 0.04). All groups improved temporally (*p* < 0.01), but RAGT+TMS yielded synergistic enhancements in functional recovery.

**Conclusion:**

This study demonstrates that combined RAGT and LF-rTMS significantly enhances post-stroke motor recovery, yielding clinically superior improvements in balance (BBS), gait symmetry, and postural control compared to RAGT alone or conventional therapy. The synergistic effects highlight TMS’s potential to augment neuroplasticity when paired with robotic training. While further large-scale trials are needed, these findings support integrating dual-modality approaches for comprehensive stroke rehabilitation.

**Clinical trial registration:**

https://www.chictr.org.cn/indexEN.html, ChiCTR2200066978.

## Introduction

Stroke constitutes a critical global health challenge, ranking as the second most common cause of mortality among non-communicative diseases with approximately 7 million deaths annually and affecting over 15 million individuals each year (1–3). Among stroke survivors, motor impairments represent the most prevalent and functionally devastating consequences, with up to 80% experiencing persistent hemiparesis that fundamentally compromise locomotion, balance, and independence in activities of daily living (4, 5). The neurophysiological basis of post-stroke motor dysfunction stems from disruptions to complex neural networks governing movement initiation, execution, and planning, including damage to critical structures such as the corticospinal tract, basal ganglia, and cerebellum (6, 7). These neurological alterations precipitate a cascade of sensorimotor deficits encompassing muscular weakness, spasticity, impaired coordination, and sensory deterioration, directly impeding patients’ capacity to generate appropriate force, control limb movements, and adapt to environmental demands during locomotion (8, 9). Consequently, stroke survivors typically exhibit reduced walking velocity, asymmetric gait patterns, heightened fall risk, and substantial challenges in community ambulation, with these impairments affecting not only physical mobility but also cognitive and emotional wellbeing (10).

Contemporary neurorehabilitation has undergone a paradigmatic evolution, transitioning from traditional compensatory approaches toward interventions that strategically leverage neuroplasticity mechanisms—the brain’s remarkable capacity for structural and functional reorganization following injury (11). This transformation has been guided by fundamental neurorehabilitation principles derived from motor learning and brain plasticity research, including task-specific practice, repetitive training, multisensory stimulation, progressive difficulty adaptation, and goal-oriented practice (12). Recent technological advances have established robotic-assisted gait training (RAGT) as an evidence-based intervention that systematically incorporates multiple neurorehabilitation principles simultaneously (13). Modern RAGT systems utilize complex exoskeleton devices and end-effector systems with increased degrees of freedom, force transmission mechanisms, and adaptive control algorithms that enable precise manipulation of gait parameters while providing intensive, standardized locomotor training (13). These systems integrate artificial intelligence, machine learning algorithms, and real-time biofeedback to customize rehabilitation procedures according to individual patient capabilities and recovery trajectories (14). The interactive nature of RAGT represents a significant advancement over conventional approaches, incorporating elements such as gaming interfaces, virtual reality environments, and sensor-based analytics to enhance patient engagement and maximize therapeutic outcomes (13, 15, 16). Recent meta-analyses demonstrate that RAGT facilitates critical sensorimotor feedback mechanisms, modifies pathological muscle activation patterns, and induces significant improvements in lower extremity function, balance, walking ability, and endurance compared to conventional rehabilitation approaches (13, 17, 18).

Parallel technological evolution in non-invasive brain stimulation has established transcranial magnetic stimulation (TMS), particularly repetitive TMS (rTMS), as a precision neuromodulation tool with demonstrated efficacy for stroke rehabilitation (19, 20). The therapeutic rationale for rTMS is grounded in the principle of interhemispheric competition, whereby stroke-induced damage creates an imbalance between hemispheric inhibitory influences, with the contra-lesional hemisphere exerting excessive inhibition over the damaged ipsilesional cortex (21, 22). Low-frequency rTMS (≤1 Hz) applied to the contra-lesional motor cortex can reduce this maladaptive hyperactivity, thereby facilitating recovery of function in the affected hemisphere and reestablishing interhemispheric balance (22, 23). Recent advancements include sophisticated coil designs incorporating theta-burst stimulation protocols, real-time computational modeling for customized dosage parameters, and neuro-navigation-guided systems that enable precise, image-based targeting of specific cortical regions (19). Current evidence suggests differential efficacy of rTMS protocols for lower extremity rehabilitation, with meta-analyses indicating that low-frequency stimulation may more effectively promote lower limb motor recovery through modulation of contra-lesional hyperactivity (24). European consensus guidelines currently recommend low-frequency rTMS as an adjunctive intervention to conventional therapy, with standardized parameters including 10–20 sessions distributed over 2–4 weeks (19).

However, despite these significant technological advances, current rehabilitation approaches face fundamental limitations that constrain optimal recovery outcomes and highlight critical gaps in therapeutic effectiveness. RAGT, while providing valuable sensorimotor input through task-specific, repetitive training, primarily targets peripheral motor systems and gait kinematics without necessarily exerting substantial direct influence on central nervous system excitability and cortical reorganization processes that are essential for lasting functional recovery (25). Evidence regarding RAGT efficacy demonstrates significant improvements in functional outcomes, with exoskeleton-type devices showing superior gains in endurance and balance measures; however, effects on critical parameters such as gait speed remain inconsistent across studies, and subgroup analyses suggest differential benefits according to stroke chronicity and individual patient characteristics (26, 27). Conversely, rTMS demonstrates clear capacity to modulate cortical excitability and facilitate neural plasticity through direct neuromodulation, but research indicates that its therapeutic effects are optimized when combined with concurrent motor training that provides the activity-dependent stimulation necessary for experience-dependent plasticity (28, 29). The theoretical foundation for combining these interventions is firmly grounded in established neurorehabilitation principles: RAGT provides essential components including task-specific practice, repetitive stimulation, multisensory feedback, and progressive difficulty adaptation, while rTMS simultaneously facilitates the neuroplasticity mechanisms through targeted cortical modulation and interhemispheric rebalancing (30). This synergistic approach aligns with fundamental motor learning theory and Hebbian principles, which emphasize that optimal neural reorganization requires both intensive peripheral sensorimotor stimulation and central nervous system priming to strengthen motor engrams, enhance corticospinal connectivity, and promote lasting adaptive plasticity (31, 32).

To address this critical gap in multimodal neurorehabilitation research, we conducted a pilot randomized controlled trial specifically investigating the combined effects of low-frequency rTMS and RAGT compared to RAGT with sham stimulation and conventional physical therapy in individuals with post-stroke hemiparesis. We systematically examined whether this theoretically driven multimodal approach would enhance gait performance, balance function, motor recovery, and postural stability by leveraging complementary neuroplasticity mechanisms through synchronized peripheral training and central neuromodulation.

This study presents comprehensive feasibility and preliminary efficacy findings from our investigation of combined RAGT and low frequency rTMS for post-stroke gait rehabilitation. We report detailed functional outcomes across multiple domains including balance, motor function, gait parameters, and postural stability, assess intervention safety and acceptability, and provide mechanistic interpretations of observed improvements based on established neuroplasticity principles. These findings contribute essential foundational evidence to the emerging field of precision neurorehabilitation by demonstrating the potential for strategically designed multimodal interventions that integrate technological and neurostimulation approaches to optimize motor recovery outcomes for stroke survivors.

## Methodology

### Study design

This study was a prospective, parallel-randomized controlled pilot trial that included stroke patients. It was approved by the ethical review committee of Chongqing Medical University, China for research involving human subjects (following Helsinki declaration) [2024 (33)], with protocol registered on the Chinese Clinical Trial Registry (Registration number: ChiCTR2200066978) before recruitment. The trial was conducted at the Department of Rehabilitation Medicine of The Second Affiliated Hospital of Chongqing Medical University (Chongqing, China), and reported in accordance with the CONSORT 2010 guidelines for randomized controlled trials (34). All Participants provided written informed consent.

### Participant’s selection

Participants were included if they met the following inclusion criteria: (1) Unilateral hemiplegic stroke; (2) Independent ambulation before onset of stroke; (3) Ability to understand and follow simple instructions; (4) Functional ambulatory category (FAC) score ≥1, i.e., (ability to walk with assistance or independently); (5) Age between 18 and 70 years. Participants were excluded based on the following exclusion criteria: (1) Unstable vital signs; (2) Severe cognitive impairments or global aphasia limiting cooperation; (3) Contraindications to rTMS, as per safety guidelines including [presence of any ferromagnetic or other metal implants in the head, neck, or upper thoracic region (e.g., intracranial aneurysm clips or coils, cochlear implants, bullet fragments), Implanted electronic medical devices (e.g., deep brain stimulators (DBS), vagus nerve stimulators (VNS), spinal cord stimulators, cardiac pacemakers or defibrillators (ICDs)), A personal or immediate family history of seizures or a diagnosis of epilepsy, Medications known to significantly lower seizure threshold, A history of significant head injury or neurosurgery, Pregnancy, lactating, or suspected pregnancy].

### Prior sample size estimation

The required sample size was estimated *a priori* based on pilot data, in which partial eta squared (η^2^) was 0.19, corresponding to a medium-to-large effect size (*f* = 0.48). Using this effect size, with a two-tailed *α* error probability of 0.05 and desired power (1 – *β*) of 0.95, the minimum required total sample size was calculated as 21 participants (7 per group). It is important to note, however, that this estimation was derived from a small pilot (*n* = 5 per group), which may have led to an optimistic effect size and consequently an underestimation of the true sample size required. Thus, although the priori calculation suggested adequacy with 7 participants per group, the actual statistical power of the present study is likely to have been lower, as reflected by the marginal significance observed in some primary outcomes. This limitation has been considered in the interpretation of the findings.

### Randomization and allocation

**Figure 1:**
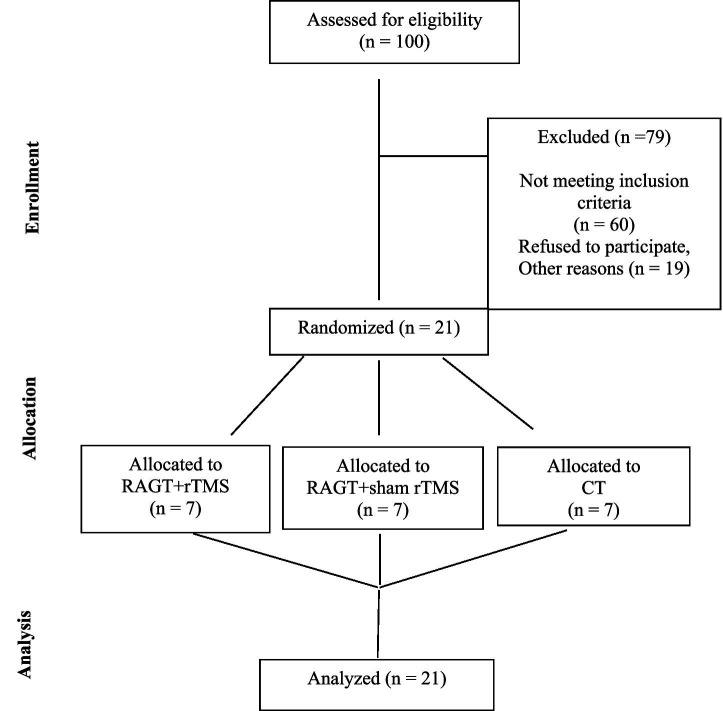
Flow diagram of participant enrollment, exclusion, randomization, allocation, and analysis.

A total of 100 individuals were assessed for eligibility between the inpatient and outpatient of rehabilitation department between November 2023 to December 2024. 79 were excluded (60 not meeting inclusion criteria, 19 declined or excluded) (Figure 1). All the included participants were allocated into three groups at a 1:1:1 ratio using a randomization tool (https://ctrandomization.cancer.gov/), by the therapist. The groups were divided based on the intervention: group RAGT+rTMS received RAGT with active low frequency rTMS; group RAGT+sham rTMS received RAGT with Sham low frequency rTMS; group CT received conventional treatment, without RAGT or rTMS. In addition to experimental therapies, each patient receives standard physiotherapy gait and balance training. All participants completed the allocated intervention and were included in the final analysis, with no dropouts or losses to follow-up. This complete retention across groups strengthens the internal validity of the trial and ensures that the outcomes are based on the originally randomized sample (intention-to-treat principle).

**Figure 2:**
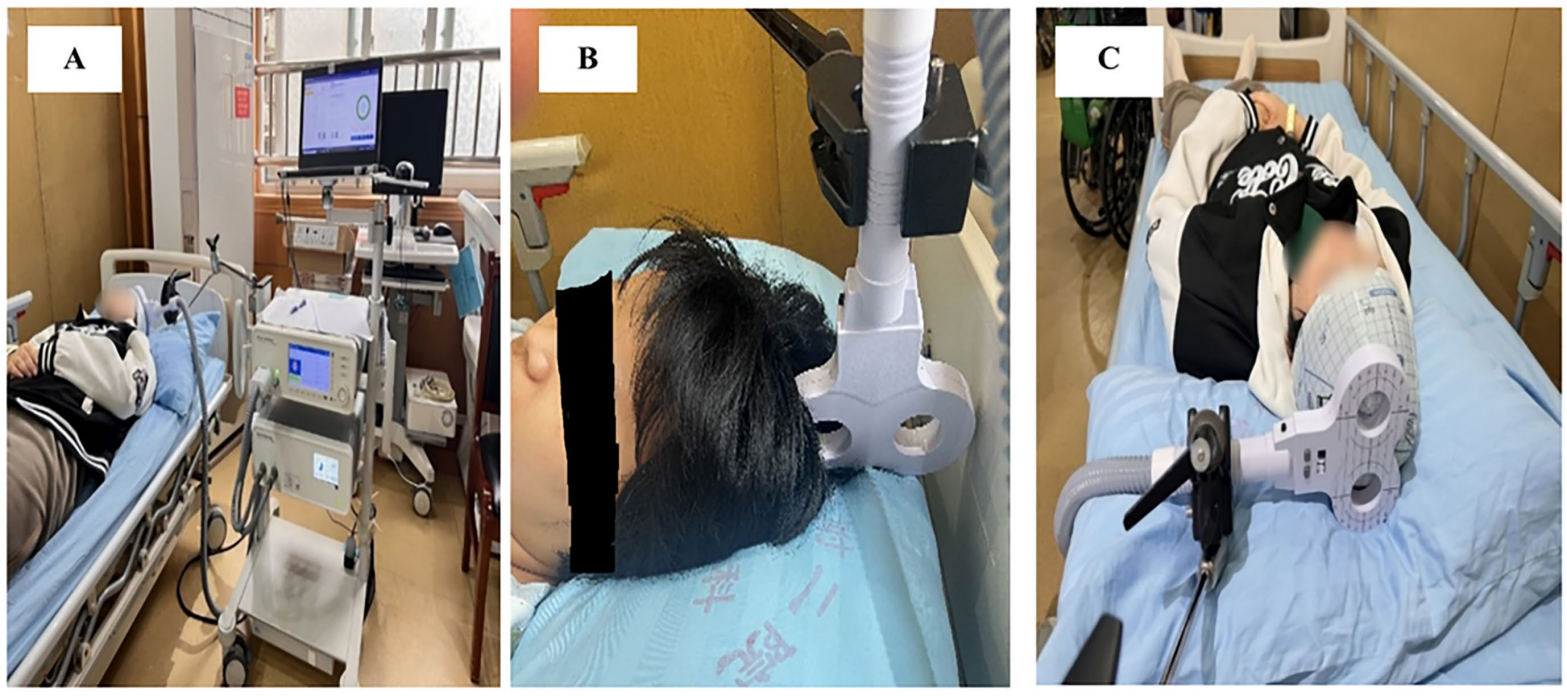
rTMS setup: **(A)** M-Series TMS system and its application; **(B)** coil positioning for sham stimulation; **(C)** coil positioning for active stimulation.

Allocation concealment was ensured by keeping the randomization list in the care of the physician (YY), who was not involved in the follow-up measurements. The main investigator (AJ, YF, IA) and patients were blinded to the allocated treatment during the entire period of data collection, while therapists remained unblinded. To maintain participant blinding, sham stimulation protocols were carefully designed: therapists positioned the coil vertically at a 90-degree angle relative to the scalp (Figure 2B) replicating the auditory and tactile sensations of active stimulation (e.g., device clicks and operational sounds) without delivering therapeutic effects (35). Participants were therefore blinded from differentiating between the real and sham stimulation.

### Interventions

Participants in this study underwent a methodical rehabilitation program that included two main interventions: repeated transcranial magnetic stimulation (rTMS) and robotic-assisted gait training (RAGT), which were administered daily for 4 weeks.

**Figure 3:**
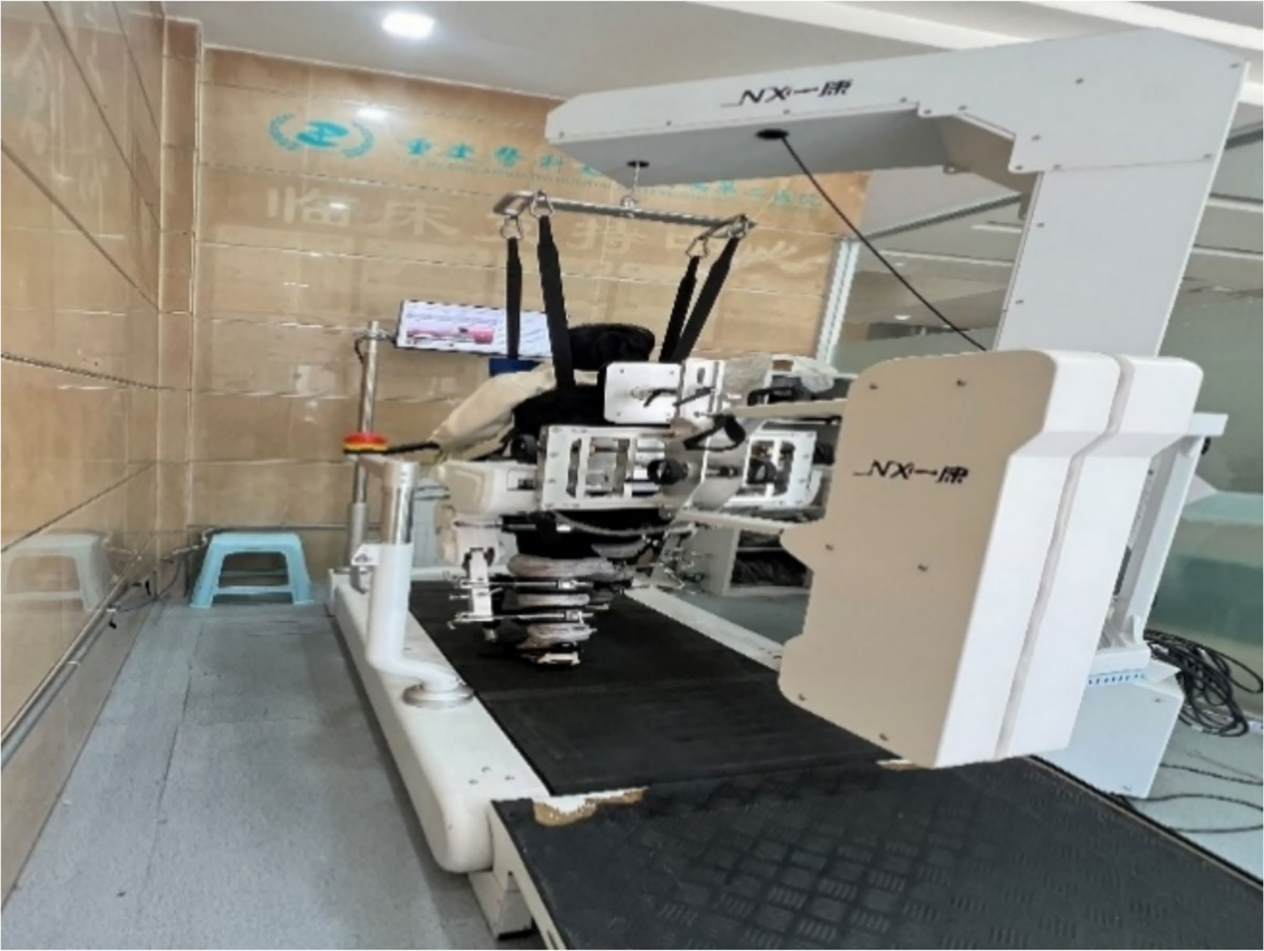
The gait training & assessment system.

Robotic assisted gait training was provided using an exoskeleton type gait robot ‘The Gait Training & Assessment System (Guangzhou Yikang Medical Equipment Industrial Co., Ltd.), consisting in a motorized exoskeleton with a treadmill, and an adjustable body-weight support (BWS) mechanism (Figure 3). The exoskeletal modules attach bilaterally to the thigh, shank and foot segments via adjustable cuffs and provide sagittal-plane actuation of the hip, knee and ankle to reproduce physiological gait kinematics. Actuation is motorized and monitored by high-resolution joint encoders and force/torque sensors to enable closed-loop control. The system’s control software runs pre-programmed normative gait templates that are scaled to participant anthropometrics and synchronized with treadmill speed; real-time feedback from joint encoders and foot-contact sensors allows the robot to operate in trajectory-tracking or adaptive “assist-as-needed” modes so that assistance can be graded according to the participant’s voluntary contribution. Mechanical support (lumbar and trunk supports, foot plates) and an emergency-stop interlock ensure participant stability and safety. Prior to each session the device was calibrated to the participant by aligning exoskeletal joint centers with anatomical landmarks, adjusting segment lengths and cuff positions, and setting assistance and BWS levels. In this trial treadmill speed ranged 1.2–1.8 km/h and BWS was titrated up to 40–60% of body weight to promote safe, symmetrical step cycles while limiting compensatory strategies.

Brain stimulation participants received concurrent 20-min rTMS sessions using the Brain Ultimate M-Series TMS System (Brain Ultimate Inc. and Shenzhen Yingchi Technology Co., Ltd.), which was equipped with a figure-8 coil (Figure 2A).

A low-frequency inhibitory protocol (0.1–1 Hz) was used to stimulate the contra-lesional primary motor cortex (M1), which corresponds to the lower-limb representation of the unaffected hemisphere, at 80% of resting motor threshold (RMT). Mechanistic and clinical studies of post-stroke motor recovery support the selection of the contra-lesional (unaffected) M1 to target interhemispheric imbalance following stroke by reducing maladaptive excitability in the non-lesioned hemisphere, thereby facilitating ipsi-lesional recovery (36). Two factors led to the selection of an 80% RMT intensity. First, sub-threshold intensities (≈80–90% RMT) are frequently used for low-frequency (≤1 Hz) inhibitory protocols in clinical rTMS trials and evidence-based guidelines because they consistently provide cortical suppression while reducing participant discomfort and direct muscle activation (37). Second, 80% RMT has been successfully used as the stimulation intensity in several randomized and controlled studies of inhibitory 1-Hz rTMS in stroke rehabilitation, demonstrating its applicability and translational value (38). Additionally, recent research shows that inhibitory effects vary in intensity, with 80% RMT balancing safety and effectiveness in human motor-cortex protocols (39). Figure-8 was positioned horizontally in a 90° mediolateral orientation over the scalp (40, 41) (Figure 2C). This orientation aligns the induced cortical current flow along the mediolateral axis (perpendicular to the interhemispheric fissure) to optimize stimulation of lower extremity (LE) motor cortex (M1). The coil was centered over the vertex and adjusted 1–2 cm laterally to target the M1 representation of the unaffected hemisphere’s lower limb musculature (40, 41).

The mediolateral coil orientation was selected to address the unique neuroanatomical challenges of LE cortical targeting (40). Optimal electric field penetration is necessary because the LE homunculus is situated deeper inside the interhemispheric fissure than upper limb representations. By aligning the transverse orientation of corticospinal axons projecting to LE muscles, medial current flow enhances the efficacy of stimulation and reduces motor thresholds (42). This orientation preferentially stimulates indirect trans-synaptic I-waves over direct D-waves for altering interhemispheric balancing and LE motor control circuitry (43).

To determine the hotspot for stimulation, Participants wore a specially made footplate while seated with the paretic ankle locked in a neutral posture. The contra lesional primary motor cortex (M1) hotspot for the tibialis anterior (TA) was found using the Brain Ultimate M-Series TMS System fitted with a figure-8 coil (Outer diameter: 75 mm, peak magnetic field: 2.3 Tesla, pulse width: 280 μs).

Coil orientation was set to induce a medial-to-lateral current flow over the interhemispheric fissure with initial placement at the vertex (Cz), then 0.5 cm lateral adjustments guided by real-time EMG feedback from the paretic TA, ipsilateral soleus, and rectus femoris (recorded via the system’s integrated 8-channel EMG module, sampling rate: 5 kHz, bandwidth: 10–500 Hz) (40, 41). Resting motor threshold (RMT) was defined as the minimum stimulator output (%MSO) required to evoke motor-evoked potentials (MEPs) ≥ 100 μV peak-to-peak amplitude in ≥5/10 trials, as validated using the device’s automated MEP detection algorithm (sensitivity: ±50 μV, latency window: 20–50 mL) (41). MEPs were recorded from the tibialis anterior (TA) muscle of the unaffected limb, corresponding to stimulation of the contralesional M1 representation of the lower extremity.

Over the course of 4 weeks, each participant completed 28 consecutive sessions, one session per day.

### Outcome measures

Primary outcomes comprised clinical measures of motor function, balance, and muscle strength. The Fugl-Meyer Assessment for lower extremity (FMA-LE) quantified motor recovery (0–34 points, maximum 34 point, with higher score representing improvement) based on reflex activity and voluntary movement capabilities, with or without synergy, coordination, and speed (44). The minimal clinically important difference (MCID) for FMA-LE in chronic post-stroke hemiparesis has been established at 6 points improvement (45). The Berg Balance Scale (BBS) evaluated functional balance through 14 activities scored 0–4 (maximum 56 points, with higher score represent improvement) (46). The minimal MCID for BBS in post-stroke hemiparesis has been established at 4–5 points (47). Manual muscle testing (MMT) of the paretic limb employed the Medical Research Council (MRC) scale (0–5) across six key muscle groups: hip flexors/extensors, knee flexors/extensors, and ankle dorsiflexors/plantar flexors. All assessments were conducted at baseline (T0) 24 h pre-intervention and post-intervention (T1) within 24 h following the final session.

Secondary outcomes encompassed objective biomechanical and functional evaluations of gait parameters and balance using validated clinical instrumentation.

**Figure 4:**
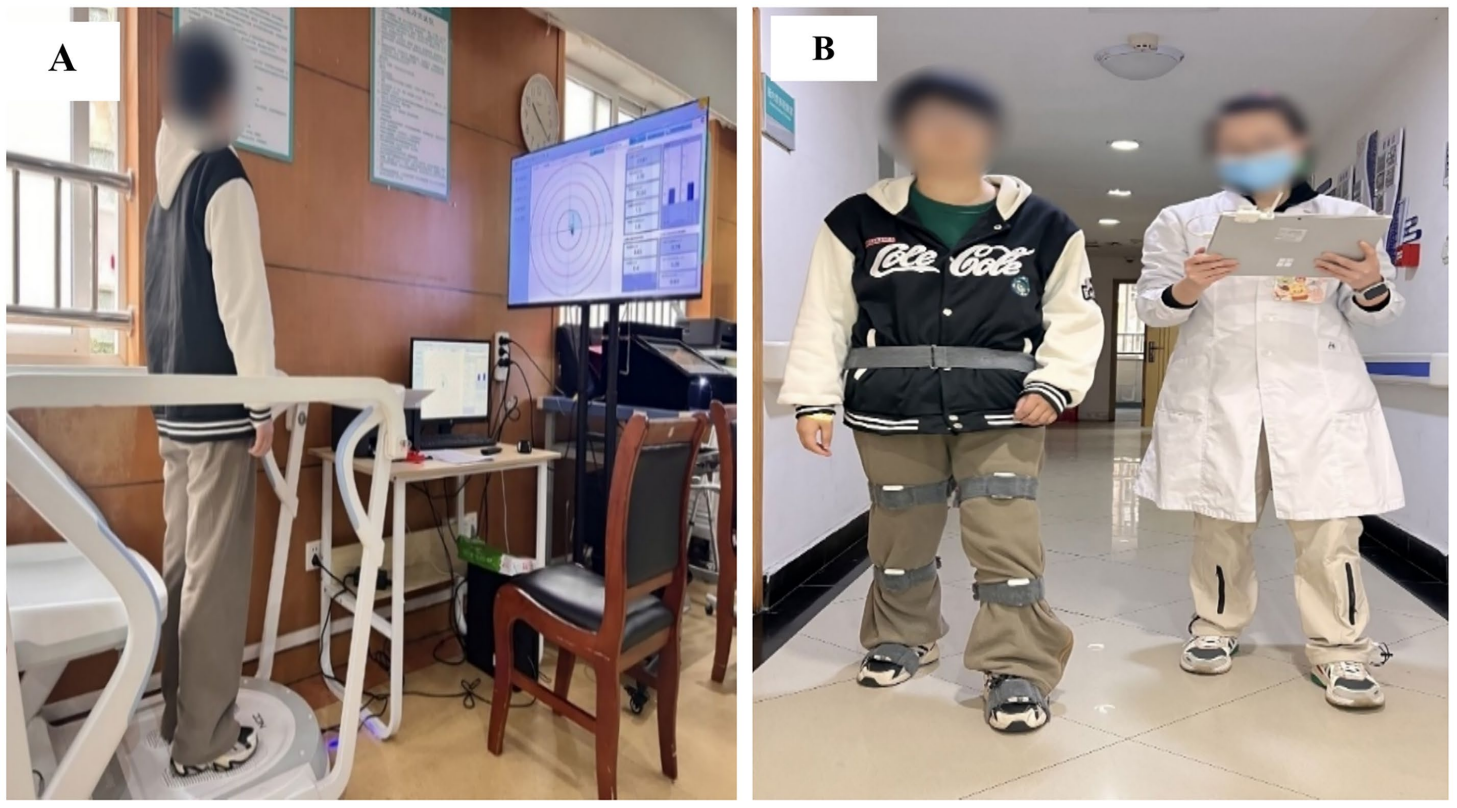
Gait analysis and balance system. **(A)** Display 3D gait analysis system and its application; **(B)** Dynamic balance assessment and training system.

Gait assessment utilized the 3D Display Gait Analysis system (Guangzhou Yeecon Medical Equipment Industrial Co., Ltd.), a wireless sensor-based platform featuring nine-axis MPU9250 inertial measurement units (IMUs) integrated into foot-mounted modules. These sensors captured triaxial acceleration (±16 g), angular velocity (±2000°/s), and geomagnetic data at 100 Hz, enabling precise quantification of spatiotemporal parameters including gait cycle duration, stride length, cadence, step symmetry, and single/double limb support time. Data transmission occurred via Bluetooth 4.0 to a handheld interface (373 mm × 335 mm × 109 mm) with subsequent processing through proprietary software (WalkAnalysis Pro v3.2), which generated three-dimensional gait reconstruction models for visualization of joint angles, foot trajectory deviations, and dynamic stability (Figure 4A).

Balance evaluation employed the Dynamic Balance Assessment and Training System (XPH-B; Jiangxi Nuo Cheng Electrical Co., Ltd.), to measure center-of-pressure (COP) trajectory, postural sway velocity (cm/s), and weight distribution asymmetry (%) during standardized protocols. The system’s software (Balance Metrics Suite v2.1) provided real-time visual feedback and quantitative data for static standing, dynamic weight-shifting, and perturbation response tasks (Figure 4B).

### Statistical analysis

Statistical analyses were conducted using SPSS software (version 26.0). The normality of data distribution was assessed using the Shapiro–Wilk test. Demographic and baseline characteristics were compared between groups using ANOVA for continuous variables and chi-square tests for categorical variables. For outcome measures, interaction effects between group and time were analyzed using mixed method ANOVA with Bonferroni *post hoc* adjustments. Within-group comparisons were illustrated using bar graphs. The level of significance was set at *p* < 0.05 (2-tailed) and was applied for all statistical tests.

## Results

Twenty-one participants with post-stroke hemiparesis were randomized into three intervention groups: robotic-assisted gait training with transcranial magnetic stimulation (RAGT+TMS; *n* = 7), RAGT with sham TMS (RAGT+shamTMS; *n* = 7), and conventional physical therapy (control; *n* = 7). Baseline demographic and clinical characteristics demonstrated (Table 1) homogeneity across all groups (*p* > 0.05), with comparable age distribution (RAGT+TMS: 54.14 ± 7.95 years; RAGT+sham TMS: 53.00 ± 12.76 years; control: 56.71 ± 20.72 years; *p* = 0.89), body mass index (23.86 ± 1.93, 25.88 ± 2.34, and 24.20 ± 2.60, respectively, *p* = 0.24), and gender distribution (*p* = 0.16). No significant between-group differences were observed in baseline motor evoked potential amplitudes (*p* = 0.56), stroke etiology (*p* = 1.00), affected hemisphere (*p* = 0.47), or chronicity (*p* = 0.54), confirming successful randomization.

**Table 1:** Characteristics of participants.

Variables		RAGT+ rTMS Group (*n* = 7)	RAGT + sham rTMS Group (*n* = 7)	Control Group (*n* = 7)	*p*
Age, y, mean (SD)		(54.14 ± 7.95)	(53.00 ± 12.76)	56.71 ± 20.72	0.89
BMI (kg/m^2^)		23.86 ± 1.93	25.88 ± 2.34	24.20 ± 2.60	0.24
Gender (male/female)		4/3	5/2	3/4	0.16
No of Sessions		28.00 ± 0.00	26.57 ± 2.99	28.00 ± 0.00	0.23
MEP		36.57 ± 21.99	46.43 ± 15.88	39.28 ± 13.54	0.56
Type of stroke					
Hemorrhagic stroke/Ischemic Stroke		4/3	4/3	4/3	1.00
Affected side (R/L)		5/2	3/4	3/4	0.47
Onset of stroke	Chronic	7	7	7	

Values are represented as mean and standard deviation and frequencies for categorical variables. RAGT+ rTMS Group indicates robotic assisted gait training plus trans magnetic stimulation, RAGT + sham rTMS Group indicates robotic assisted gait training plus sham trans-magnetic stimulation; Control Group indicate conventional physical therapy. MEP (motor evoke potential). Between group differences were calculated using ANOVA for continuous, and chi-square test for categorical variables, *p* < 0.05 significant.

### Primary outcomes (balance and motor function)

Mixed-model ANOVA with Bonferroni correction demonstrated robust time effects across the primary clinical measures (Berg Balance Scale [BBS], Fugl-Meyer Assessment [FMA], and Manual Muscle Testing [MMT]; all *p* < 0.001), indicating clinically meaningful improvement from baseline to post-intervention across the cohort (Table 2). Group main effects were non-significant for BBS, FMA and MMT (*p* = 0.68, 0.66 and 0.62, respectively), while group × time interactions reached statistical significance for BBS (*p* = 0.05) and FMA (*p* = 0.04), but not MMT (*p* = 0.10), indicating differential magnitudes of change between intervention arms over time (Table 2).

**Table 2:** Result of for primary outcome measure with mixed method ANOVA with Bonferroni correction.

Group	Time	Primary outcome measure		
		BBS	FMA	MMT
RAGT+ rTMS Group (*n* = 7)	T1	25.42 ± 16.97	23.28 ± 8.90	16.71 ± 7.99
	T2	48.00 ± 8.74	29.14 ± 6.91	23.71 ± 6.05
	Change (T2−T1)	22.58	5.86	7
RAGT+ sham rTMS Group (*n* = 7)	T1	23.40 ± 21.70	21.20 ± 12.59	16.00 ± 6.24
	T2	38.80 ± 14.04	23.20 ± 11.64	19.60 ± 5.59
	Change (T2−T1)	15.4	1.61	3.6
Control Group (*n* = 7)	T1	34.00 ± 14.62	22.00 ± 4.54	16.43 ± 4.35
	T2	41.85 ± 11.11	24.28 ± 4.34	18.14 ± 4.91
	Change (T2−T1)	7.85	2.28	1.71
*p*	Time	0.001	0.001	0.001
	Group	0.68	0.66	0.62
	Time*Group	0.05	0.04	0.10

Values are represented as mean and standard deviation. RAGT+ sTMS Group indicates robotic assisted gait training plus trans magnetic stimulation, RAGT + sham rTMS Group indicates robotic assisted gait training plus sham trans-magnetic stimulation; Control Group indicate conventional physical therapy. Between group differences were calculated using mixed method ANOVA, Changes between T1 and T2 (Change (T2−T1)) were calculated as the difference in means and standard deviations (T2 − T1), *p* < 0.05 significant.

**Figure 5:**
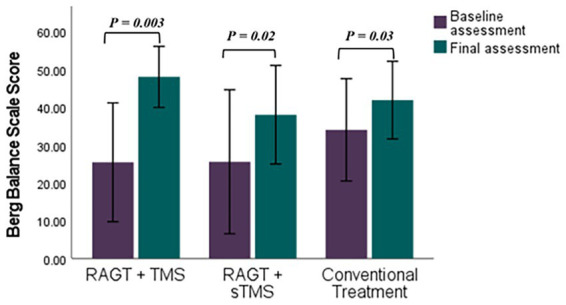
Comparative efficacy of RAGT combined with TMS or sTMS versus conventional treatment on Berg Balance Scale scores: baseline to final assessment analysis. *p* value calculated using paired sample t-test, *p* < 0.05 is significant.

**Figure 6:**
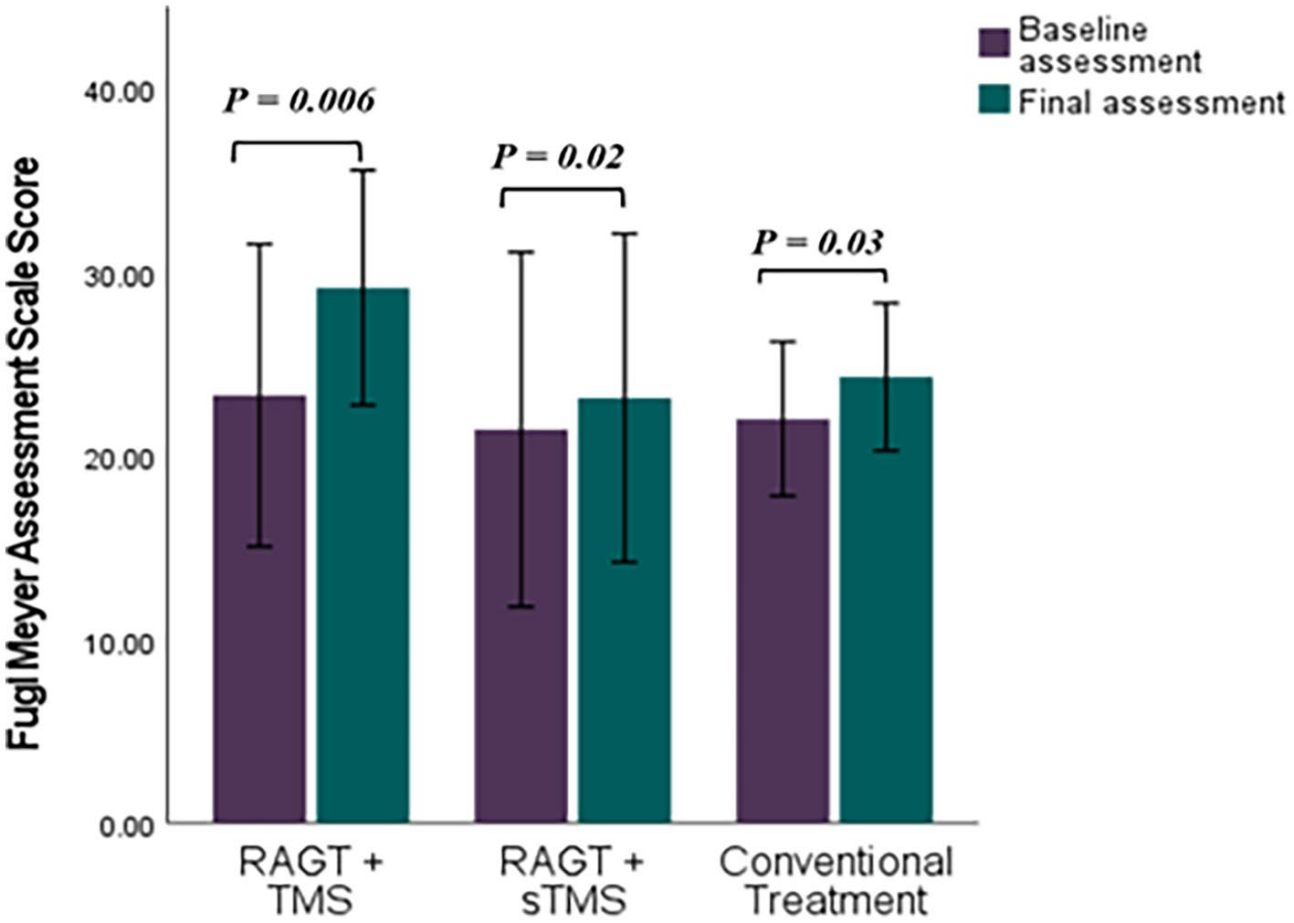
Comparative efficacy of RAGT combined with TMS or sTMS versus conventional treatment on Fugl Meyer Assessment Scale scores: baseline to final assessment analysis. *p* value calculated using paired sample test, *p* < 0.0.5 is significant.

**Figure 7:**
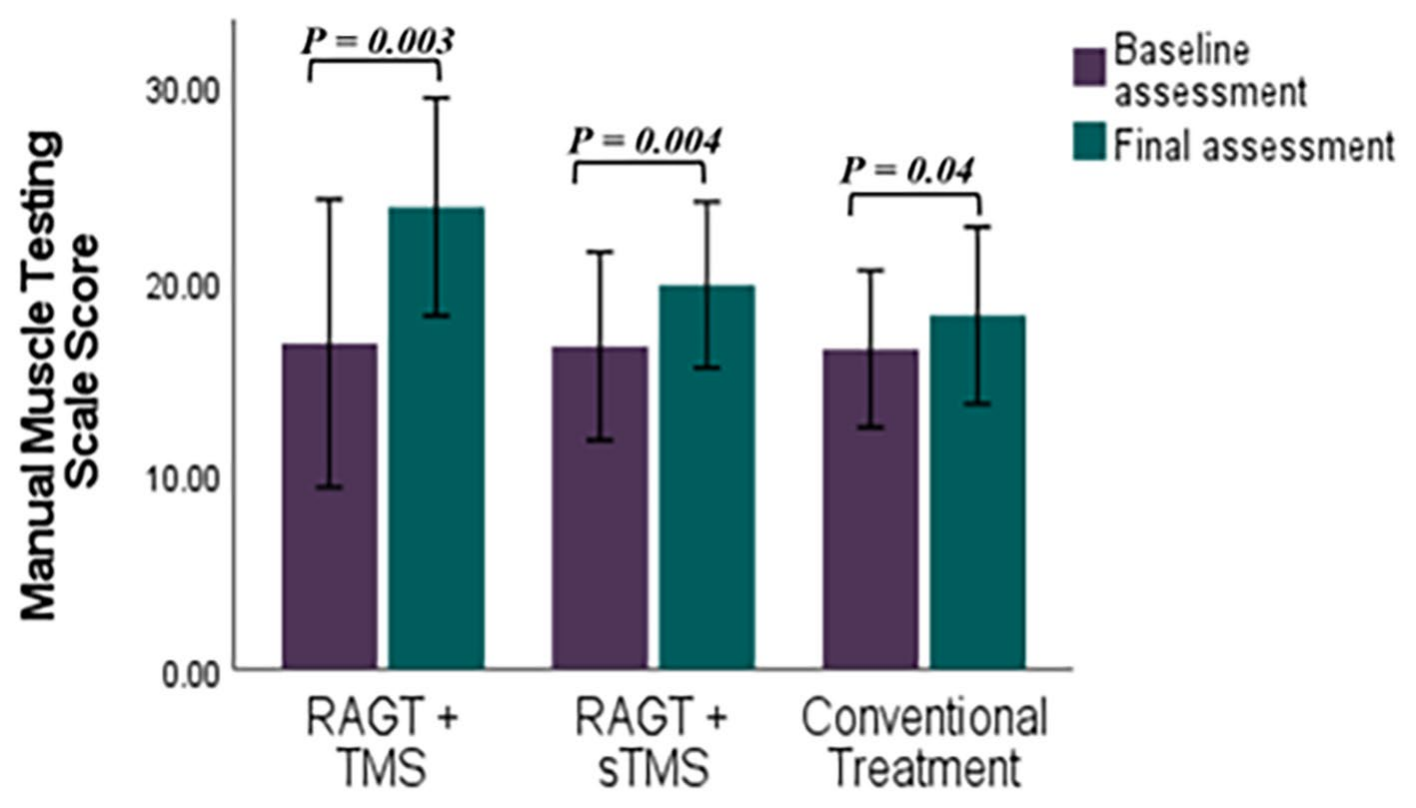
Comparative efficacy of RAGT combined with TMS or sTMS versus conventional treatment on Manual Muscle Testing Scale scores: baseline to final assessment analysis. *p* value calculated using paired sample test, *p* < 0.0.5 is significant.

Mean (±SD) changes from T1 to T2 were larger in the RAGT+rTMS group than in the RAGT+sham and control groups: BBS change *Δ* = +22.58 points (from 25.42 ± 16.97 to 48.00 ± 8.74) versus +15.40 and +7.85 in RAGT+sham and control, respectively; FMA change Δ = +5.86 points (23.28 ± 8.90 to 29.14 ± 6.91) versus +1.61 and +2.28; and MMT change Δ = +7.00 points (16.71 ± 7.99 to 23.71 ± 6.05) versus +3.60 and +1.71 (Figures 5–7). These numerical differences, together with the observed group × time interactions for BBS and FMA, are consistent with greater balance and motor gains in the RAGT+rTMS arm; however, between-group comparisons at individual timepoints were not statistically significant.

### Secondary outcomes — three-dimensional gait analysis

Computerized gait analysis revealed significant time-dependent improvements across the majority of spatiotemporal parameters (time effects *p* ≤ 0.01 for the reported variables), indicating overall improvement in gait performance for participants across interventions (Table 3). Group × time interaction was statistically significant only for left step length (*p* = 0.04). Notably, the control group showed the largest mean increase in left step length (*Δ* = +12.00 ± 6.26 cm; 51.85 ± 16.86 to 63.85 ± 10.60), compared with RAGT+rTMS (Δ = +6.86 ± 0.59 cm; 43.71 ± 12.24 to 50.57 ± 11.65) and RAGT+sham (Δ = +1.14 ± 0.57 cm). Right step length increased uniformly across groups (mean increases ≈ + 11.00–11.28 cm; time effect *p* = 0.001; group *p* = 0.96).

**Table 3:** Result of for secondary outcome measure (computerized 3D gait analysis variables) with mixed method ANOVA with Bonferroni correction.

Group	Time	Computerized gait variables										
		Step length left leg (cm)	Step length right leg (cm)	Gait cycle (sec)	Step frequency (steps/min)	Gait speed (meter/s)	Swing phase (%) left leg	Swing phase (%) right leg	Supporting phase (%) left leg	Supporting phase (%) right leg	Dual support (%) initial phase	Dual support (%) terminal phase
RAGT+ rTMS Group (*n* = 7)	T1	43.7 ± 12.2	34.7 ± 13.4	3.7 ± 1.1	69.3 ± 17.9	0.2 ± 0.9	30.1 ± 10.3	26.9 ± 14.5	69.0 ± 11.3	68.5 ± 16.5	19.9 ± 7.8	25.2 ± 9.1
	T2	50.5 ± 11.6	45.7 ± 12.2	2.7 ± 0.7	83.6 ± 14.5	0.4 ± 0.1	36.3 ± 9.6	34.5 ± 12.9	63.1 ± 10.8	63.2 ± 14.8	14.4 ± 5.8	18.6 ± 10.1
RAGT+ sham rTMS Group (*n* = 7)	T1	56.7 ± 23.8	36.4 ± 22.6	3.3 ± 0.9	77.3 ± 20.3	0.3 ± 0.2	26.1 ± 11	27.8 ± 14.9	73.9 ± 11	72.2 ± 14.9	14.8 ± 13.9	13.4 ± 7.3
	T2	57.8 ± 24.3	46.1 ± 20.1	2.6 ± 1.0	85.2 ± 20.3	0.3 ± 0.2	28.1 ± 10.8	32.5 ± 16.2	71.9 ± 10.6	67.7 ± 16.1	13.7 ± 14.1	11.6 ± 7.5
Control Group (*n* = 7)	T1	51.8 ± 16.7	40.1 ± 21.3	3.1 ± 1.0	85.3 ± 16.6	0.3 ± 0.1	25.5 ± 11.5	28.2 ± 8.7	74.6 ± 11.4	71.8 ± 8.7	22.0 ± 11.6	23.9 ± 6.4
	T2	63.8 ± 10.6	51.4 ± 15.6	2.8 ± 1.1	91.6 ± 21.2	0.4 ± 0.1	28.6 ± 10.2	31.9 ± 8.6	71.3 ± 10.4	71.2 ± 8.8	17.2 ± 9.4	19.8 ± 8.4
*p*	Time	0.001	0.001	0.001	0.001	0.001	0.001	0.001	0.001	0.003	0.001	0.001
	Group	0.43	0.81	0.85	0.46	0.57	0.46	0.99	0.40	0.72	0.64	0.06
	Time*Group	0.04	0.96	0.11	0.37	0.10	0.18	0.42	0.24	0.14	0.18	0.18

Data are presented as mean ± standard deviation (SD), and statistical analysis was performed using mixed method ANOVA to evaluate the effects of Time (T1 vs. T2), Group, and their interaction (Time*Group) on computational balance variables, with *p*-values indicating the level of significance (*p* < 0.05 denoting statistical significance). [Step Length; Symmetrical step lengths closer to the reference range reflect better propulsion and balance], [Gait Cycle; Cycles returning to the reference range (e.g., from 1.3 s to 1.1 s) suggest normalized pacing], [Step Frequency; Increased step frequency within the reference range signals enhanced mobility and confidence], [Gait Speed; Increased speed within a functional range enhances independence], [Swing Phase (%) Longer swing phases (e.g., from 30 to 40%) allow adequate leg clearance], [Supporting Phase; Shorter support phases (e.g., from 70 to 60%) indicate better confidence and speed], [Dual support; Reduced dual support (e.g., from 20 to 12%) reflects improved balance].

Temporal gait parameters showed numerically greater improvements in the RAGT+rTMS arm — for example, a larger reduction in gait cycle duration (−1.03 ± 0.32 s in RAGT+rTMS vs. − 0.32 ± 0.05 s in controls), a greater increase in step frequency (*Δ* = +14.26 ± 3.34 steps/min), reductions in dual support phases (initial *Δ* = −5.44 ± 2.01%; terminal *Δ* = −6.62 ± 1.06%), and improved swing-phase symmetry (left Δ = +6.16 ± 0.71%; right *Δ* = +7.59 ± 1.65%). These temporal changes did not reach significance for between-group comparisons (all group or time × group *p* > 0.05).

### Secondary outcomes — computational balance analysis

Computational balance assessment revealed (Table 4) significant improvements in all parameters over time (all *p* ≤ 0.006), with significant group-by-time interactions for *X*-axis center of gravity offset (*p* = 0.04) and average shaking speed (*p* = 0.04). The RAGT+TMS group demonstrated superior reductions in postural instability metrics, including total oscillation trajectory length (*Δ* − 25.01 ± 17.02 cm; 48.27 ± 23.64 to 23.26 ± 6.66), envelope area (Δ − 1.93 ± 1.03 cm^2^), and X-axis offset (Δ − 2.72 ± 1.96 cm). These improvements substantially exceeded those observed in RAGT+sham TMS (oscillation length: Δ − 9.57 ± 2.11 cm) and control groups (Δ − 16.21 ± 2.68 cm). Further, the RAGT+TMS cohort exhibited significant reductions in average shaking speed (Δ − 0.70 ± 0.63 cm/s) and track length per unit area (Δ − 2.91 ± 2.14 cm/cm^2^), compared to minimal changes in the RAGT+sham TMS (Δ − 0.11 ± 0.08 cm/s) and control groups (Δ − 0.12 ± 0.03 cm/s).

**Table 4:** Result of for secondary outcome measure (computerized 3D balance analysis variables) with mixed method ANOVA with Bonferroni correction.

Group	Time	Computational balance variables					
		Total oscillation trajectory length (cm)	Envelope area (cm2)	*X*-axis center of gravity offset (cm)	*Y*-axis center of gravity offset (cm)	Average shaking speed (cm/s)	Track length per unit area (cm/cm^2^)
RAGT+ rTMS Group (*n* = 7)	T1	48.27 ± 23.64	3.28 ± 1.90	4.52 ± 3.59	1.61 ± 1.10	1.63 ± 0.82	9.97 ± 6.32
	T2	23.26 ± 6.66	1.35 ± 0.87	1.80 ± 1.63	0.74 ± 0.35	0.93 ± 0.19	7.06 ± 4.18
	Change (T2−T1)	-25.01 ± 17.02	−1.93 ± 1.03	−2.72 ± 1.96	−0.87 ± 0.75	−0.70 ± 0.63	−2.91 ± 2.14
RAGT+ sham rTMS Group (*n* = 7)	T1	36.14 ± 14.17	5.12 ± 2.56	2.97 ± 1.95	1.53 ± 1.08	1.20 ± 0.47	7.78 ± 2.40
	T2	26.57 ± 16.28	3.18 ± 1.48	2.29 ± 1.45	1.24 ± 0.96	1.09 ± 0.55	5.89 ± 2.37
	Change (T2−T1)	−9.57 ± 2.11	−1.94 ± 1.08	−0.68 ± 0.50	−0.29 ± 0.12	−0.11 ± 0.08	−1.89 ± 0.03
Control Group (*n* = 7)	T1	37.51 ± 11.45	4.75 ± 2.04	2.10 ± 2.05	1.15 ± 0.71	1.10 ± 0.14	7.68 ± 4.32
	T2	21.30 ± 8.77	3.43 ± 1.81	1.46 ± 2.06	0.78 ± 0.34	0.98 ± 0.11	5.02 ± 2.60
	Change (T2−T1)	−16.21 ± 2.68	−1.32 ± 0.23	−0.64 ± 0.01	−0.37 ± 0.37	−0.12 ± 0.03	−2.66 ± 1.72
*p*	Time	0.001	0.001	0.001	0.001	0.006	0.001
	Group	0.58	0.08	0.47	0.60	0.52	0.54
	Time*Group	0.0.29	0.78	0.04	0.19	0.04	0.75

Data are presented as mean ± standard deviation (SD), and statistical analysis was performed using mixed method ANOVA to evaluate the effects of Time (T1 vs. T2), Group, and their interaction (Time*Group) on computational balance variables, with *p*-values indicating the level of significance (*p* < 0.05 denoting statistical significance); changes between T1 and T2 (Change (T2−T1)) were calculated as the difference in means and standard deviations (T2 − T1), where negative values indicate a reduction in the respective variable from T1 to T2 and positive values indicate an increase. [Total Oscillation Trajectory Length; A decrease in this value indicates reduced sway and better balance control], [Envelope area; A smaller area signifies more precise COG control], [*X* and *Y*-axis center of gravity offset; Shorter lengths in both axes suggest reduced unnecessary movement], [Average shaking speed; Lower speeds suggest reduced unnecessary movement and enhanced stability], [Track length per unit area; A decrease indicates more efficient COG movement].

### Summary of primary patterns

Across outcomes, all three groups exhibited significant within-subject improvements over time. The RAGT+rTMS arm showed consistently larger mean changes in balance, motor and many computational balance metrics, and group × time interactions for selected measures (BBS, FMA, X-axis offset, average shaking speed) support a differential effect of the combined intervention. Nevertheless, many between-group comparisons did not reach statistical significance; given the small group sizes and observed variance, these results should be interpreted cautiously.

## Discussion

This pilot study provides preliminary evidence suggesting that integrating robotic-assisted gait training (RAGT) with transcranial magnetic stimulation (TMS) may yield enhanced improvements in post-stroke rehabilitation compared to RAGT with sham TMS or conventional therapy. The RAGT+TMS group demonstrated trends toward superior gains in balance (22.58-point increase in BBS), motor function (5.86-point FMA improvement), gait speed (0.23–0.35 m/s), and postural stability (51.81% reduction in oscillation trajectory length). While between-group differences did not achieve statistical significance in this pilot investigation, these findings suggest potential synergistic effects that align with evidence supporting the combination of neuromodulation and task-specific training, particularly in enhancing corticospinal plasticity and functional recovery (48, 49). The differential trends between RAGT+TMS and RAGT+sham TMS groups underscore the potential importance of active neuromodulation, contrasting with previous studies such as Edwards et al.’s findings of no significant differences between tDCS and sham groups during robot-assisted arm training (50), and Picelli et al. (51) who reported no additional benefits when combining tDCS with spinal stimulation during RAGT. Although not statistically significant between-group differences were observed for the primary or secondary outcomes, the numerically superior improvements in the RAGT+TMS group compared with RAGT+sham and control suggest a potential additive effect of TMS in enhancing neuroplasticity. These findings should be interpreted cautiously, given the limited sample size and pilot nature of this investigation, but they nonetheless point toward promising trends warranting further investigation in larger, adequately powered trials.

The mechanisms underlying these observed trends may relate to the complementary neuroplasticity effects of combined interventions. Unlike tDCS or intermittent theta-burst stimulation (iTBS), low-frequency TMS provides focal modulation of corticospinal pathways, potentially enhancing synaptic efficacy and cortical activation in the ipsilesional hemisphere while reducing maladaptive contra-lesional hyperactivity (33, 49). This targeted neuromodulation, when combined with the intensive, task-specific sensorimotor stimulation provided by RAGT, may create optimal conditions for experience-dependent plasticity by simultaneously addressing central nervous system excitability and peripheral motor training demands.

The pronounced trends toward improved postural stability observed in our RAGT+TMS group differentiate it from conventional therapies and align with previous research demonstrating enhanced outcomes when combining robotic technology with neuromodulation. Shema et al., and Rikhof et al., noted that combinations of robotic technology and electrical stimulation yield superior outcomes compared to robotic training alone (52, 53). Though their focus was on electrical stimulation rather than TMS, the principle of combining peripheral and central neuromodulation aligns with our rationale for integrating RAGT with TMS. Additionally, Naro et al. underscored in their scoping review that NIBS paired with robotic rehabilitation may be most effective when interventions are tailored to individual neurophysiological profiles (54), a consideration that future studies could incorporate by personalizing TMS parameters based on baseline MEP amplitudes or lesion topography.

Importantly, the lack of significant interactions in right step length or swing phase symmetry (*p* > 0.05) suggests that TMS’s effects may be lateralized and context-dependent, reinforcing the need for targeted stimulation protocols. This observation parallels the heterogeneity in outcomes noted by Wang et al., depending on stimulation site and protocol duration (55). This aligns with studies showing that TMS protocols targeting the ipsilesional motor cortex may yield differential functional gains compared to non-focal methods (33, 56), Research demonstrates that rTMS coupled with motor training can increase ipsilesional MEP amplitudes and improve movement symmetry in stroke patients (56). Similarly, Le Dai et al. (49) reported that iTBS combined with robotic upper limb training not only improved FMA scores but also shifted cortical activation to the affected hemisphere, as measured by functional near-infrared spectroscopy (fNIRS). These findings suggest that TMS may enhance the brain’s capacity for reorganization by promoting ipsilesional cortical activation, a critical factor in functional recovery.

The role of MEPs as biomarkers of corticospinal integrity provides additional mechanistic insights. In our study, while baseline MEP amplitudes did not differ between groups (*p* = 0.56), the RAGT+TMS group exhibited pronounced functional trends, suggesting that TMS may have augmented neural responsiveness to training. This mirrors findings from Buetefisch et al.’s work (57), where synchronous TMS and motor training in healthy individuals enhanced motor learning through LTP (long-term potentiation)-like plasticity mechanisms (57).

RAGT provides repetitive, sensorimotor-enriched input that potentially reinforces synaptic plasticity through Hebbian learning principles (“cells that fire together, wire together”) (58). When synchronized with TMS, this process may be amplified, as the combined intervention theoretically pairs corticospinal activation with task-specific movement patterns. Additionally, the repetitive nature of RAGT alone may explain the significant time-dependent improvements observed across all groups in our study (e.g., significant within-group improvements in BBS, FMA, and gait parameters, *p* < 0.001). These findings align with Edwards et al. who reported clinical improvements in chronic stroke patients undergoing intensive robot-assisted arm training, even in the absence of significant tDCS effects (50). This suggests that repetitive, task-oriented training inherently drives plasticity, although potentially to a lesser degree than when combined with targeted neuromodulation.

The lateralized improvement trend in left step length (Δ6.86 cm, *p* = 0.04) observed in the RAGT+TMS group suggests that TMS may preferentially enhance neural drive to the affected hemisphere, potentially counteracting interhemispheric inhibition—a phenomenon where the unaffected hemisphere suppresses activity in the lesioned hemisphere, exacerbating motor deficits (59). This mechanism is further supported by Veldema and Gharabaghi, who identified bilateral or ipsilesional stimulation as potentially more effective than unilateral approaches for improving gait and balance (33). In contrast, the uniform non-significant improvement in right step length across all groups (*p* = 0.96 for interaction) likely reflects compensatory strategies mediated by the less-impaired hemisphere, a common adaptation in post-stroke gait as noted by Morone et al., (60). The differential effects on left vs. right step length underscore the potential importance of modality-specific neuromodulation. While RAGT+sham TMS and conventional therapy groups may have relied on compensatory mechanisms, the RAGT+TMS group showed trends toward achieving recovery by potentially restoring neural control to the affected limb. This aligns with Naro et al. (54) who emphasized that NIBS interventions should be tailored to individual neurophysiological profiles to optimize outcomes.

Notably, all groups exhibited significant time-dependent improvements in balance, motor function, and gait parameters (*p* < 0.001 for time effects), reflecting the inherent benefits of structured, repetitive training present in both RAGT and conventional therapy. For example, Picelli et al. (51) observed improvements in gait velocity across all groups undergoing robot-assisted training, even when combined with sham stimulation. These overall improvements also highlight the role of natural biological recovery processes in stroke patients. However, the trends toward greater gains in the RAGT+TMS group (*p* < 0.05 for some group-by-time interactions) suggest that TMS may accelerate and amplify these natural recovery processes, though this interpretation requires confirmation in larger studies.

### Clinical implication

This study represents one of the first blinded, three-arm pilot randomized controlled trials to investigate the combined effects of RAGT and low-frequency TMS on stroke rehabilitation using a multi-modal outcomes approach. The experimental group achieved a 5.86-point gain in FMA-LE scores, aligning with the established minimal clinically important difference (MCID) of nearly 6 points for chronic hemiparesis. Similarly, BBS scores improved by 22.58 points, surpassing the MCID threshold of 4–5 points (42), highlighting the intervention’s efficacy in functional balance recovery. These results validate RAGT+TMS as a substantial strategy to address gait asymmetry, postural instability, and corticospinal dysfunction.

Clinicians should prioritize dynamic balance interventions, such as postural stability assessments and asymmetric gait training (e.g., split-belt treadmills), to mitigate fall risk and counteract compensatory strategies. The lateralized improvement in step length (Δ6.86 cm) highlights TMS’s capacity to enhance ipsi-lesional neural drive, promoting true recovery overcompensation. Personalizing TMS protocols based on neurophysiological profiles (e.g., lesion location, corticospinal excitability) is critical, high-frequency stimulation may benefit patients with residual ipsilesional connectivity, while contra-lesional inhibition could address interhemispheric imbalance. Additionally, structured, high-dose rehabilitation integrating cortical challenges (e.g., obstacle negotiation, dual-task training) and neuromodulation should be prioritized in subacute/chronic populations to optimize neuroplasticity and functional gains. This study highlights the potential of combining robotic-assisted gait training with non-invasive brain stimulation to enhance neuroplasticity-guided rehabilitation. Such strategies support clinician training (SDG 4) (61), advance accessible and adaptive rehabilitation technologies (SDGs 3 and 9) (62, 63), and help reduce inequities in access to innovative care (SDG 10) (64). Collectively, these approaches may improve functional recovery, quality of life, and ultimately reduce stroke-related morbidity and mortality.

### Limitations and future directions

Despite rigorous randomization and baseline homogeneity, several limitations must be acknowledged, the small cohort (*n* = 21) and single-center design, limits statistical power and generalizability, larger trials are needed to validate these findings (60). Secondly, while the study demonstrates significant improvements in FMA-LE and BBS scores, these findings may not fully reflect the broader stroke population, especially those with varying degrees of impairment or those in different stages of rehabilitation. Additionally, the lack of diversity in the sample limits the applicability of the findings to non-Chinese populations, whose neurophysiological profiles and rehabilitation needs may differ. Lastly, the absence of long-term follow-up data precludes conclusions about sustained benefits.

Future research should investigate hybrid interventions combining TMS with other NIBS modalities (e.g., transcranial alternating current stimulation [tACS]) to target multiple nodes of the motor network, integrate kinematic and neurophysiological biomarkers (e.g., MEPs, cortical activation maps) to personalize rehabilitation, and conduct cost-effectiveness analyses to evaluate the long-term economic impact of RAGT+TMS compared to conventional therapies.

## Conclusion

This pilot randomized controlled trial evaluated the feasibility and preliminary efficacy of combining robotic-assisted gait training with low-frequency transcranial magnetic stimulation for post-stroke motor rehabilitation. The combined intervention produced trends toward enhanced recovery across multiple functional domains versus controls, but between-group differences did not reach statistical significance in this small, exploratory sample, suggesting a possible synergistic effect that requires larger, adequately powered trials. The approach is theoretically well supported by motor-learning and neuroplasticity principles: task-specific robotic training provides intensive peripheral sensorimotor input while targeted cortical stimulation modulates central excitability. These results demonstrate feasibility and recommend that future work focus on larger samples, longer interventions, mechanistic studies, and standardized protocols to determine whether multimodal neurorehabilitation can meaningfully improve outcomes and be translated into practice.

## Data Availability

The raw data supporting the conclusions of this article will be made available by the reasonable request from the first or correspondent authors.
